# Supplementary material for: Functional characterization of RebL1 highlights the evolutionary conservation of oncogenic activities of the RBBP4/7 orthologue in Tetrahymena thermophila
Source: Nucleic Acids Res. 2021 Jun 4;49(11):6196–212. doi: 10.1093/nar/gkab413 (PMC8216455; doi:10.1093/nar/gkab413)
Supplement: gkab413_Supplemental_Files [file gkab413_supplemental_files.zip › Supplemental methods RebL1 April 2021.pdf]

Functional characterization of RebL1 highlights the evolutionary conservation of oncogenic activities of the RBBP4/7 orthologue in *Tetrahymena thermophila*

Syed Nabeel-Shah[1,9], Jyoti Garg[1,2,8], Alejandro Saettone[1,8], Kanwal Ashraf [2], Hyunmin Lee[3,4], Suzanne Wahab[1], Nujhat Ahmed[4,5], Jacob Fine[2], Joanna Derynck[1], Shuye Pu[4], Marcelo Ponce[6], Edyta Marcon[4], Zhaolei Zhang[3,4,5], Jack F Greenblatt[4,5], Ronald E Pearlman[2], Jean-Philippe Lambert[7,*] and Jeffrey Fillingham[1, *]

[1.] Department of Chemistry and Biology, Ryerson University, 350 Victoria St., Toronto M5B 2K3, Canada.
[2.] Department of Biology, York University, 4700 Keele St., Toronto, M3J 1P3, Canada.
[3.] Department of Computer Sciences, University of Toronto, Toronto, M5S 1A8, Canada.
[4.] Donnelly Centre, University of Toronto, Toronto, M5S 3E1, Canada.
[5.] Department of Molecular Genetics, University of Toronto, Toronto, M5S 1A8, Canada.
[6.] SciNet HPC Consortium, University of Toronto, 661 University Avenue, Suite 1140, Toronto, M5G 1M1, Canada.
[7.] Department of Molecular Medicine, Cancer Research Center, Big Data Research Center, Université Laval, Quebec city, Canada; CHU de Québec Research Center, CHUL, 2705 Laurier Boulevard, Quebec city, G1V 4G2, Canada.
[8.] These authors contributed equally to this work
[9.] Present address: Donnelly Centre, University of Toronto, Toronto, M5S 3E1, Canada. Department of Molecular Genetics, University of Toronto, Toronto, M5S 1A8, Canada.

[*] Corresponding authors: jeffrey.fillingham@ryerson.ca; Jean-Philippe.Lambert@crchudequebec.ulaval.ca

Supplementary Methods

Molecular evolutionary analyses

To search the *Tetrahymena* genome for putative orthologs, we used amino acid sequences of human MuvB subunits, Lin9, Lin 37, Lin 52, Lin 54 and RBBP4. Protein sequences retrieved were analyzed at the Pfam (http://pfam.sanger.ac.uk/) (1) and SMART (http://smart.embl-heidelberg.de/) (2) databases to examine the domain architecture. To examine the % identity and sequence homology, multiple sequence alignment analysis was carried out using UniProtKB (https://www.uniprot.org/align/).

All phylogenetic analyses were conducted using protein sequences of identified orthologs. Multiple sequence alignments were constructed using MUSCLE with default parameters. All

protein phylogenetic analyses were carried out using the neighbour-joining method under p-distances using MEGA 7 (3). The reliability of the resulting phylogenetic trees was assessed using the bootstrap method (1000 replicas for each tree). The structural prediction and superimposition were carried out using I-TASSER server (4).

Generation of whole cell extracts and Western blotting

10% trichloroacetic acid (TCA) was used to prepare whole-cell extracts (WCE). The WCE was incubated on ice for 20-30 min. 100µL of SDS loading dye and 10µL of 1N NaOH was added to neutralize the solution. WCEs were electrophoresed through 10% SDS-PAGE and proteins were transferred to nitrocellulose. After blocking in 5% skim milk, membrane was probed with the antibodies indicated. Antibodies and dilutions used were anti-Flag (1:5000; Sigma), anti-Actin (1:10000; Abcam).

Indirect immunofluorescence

Growing *Tetrahymena* cells were fixed to perform indirect immunofluorescence as previously described (5). Briefly, cells were fixed with 0.34% Schaudinn's fixative (2:1 ration of saturated $HgCl_2$ [Sigma-Aldrich]: ethanol) and cold methanol was used for membrane-permeabilization on ice for 10 min. Cells were washed with PBS (4.3 mM Na_2HPO_4, 1.47 mM KH_2PO_4, 137 mM NaCl, 2.7 mM KCl, pH 7.4), incubated with Rhodamine conjugated secondary Rabbit anti mouse IgG antibody (1:1000; Jackson Immuno Research Laboratories, Inc, RRID: AB_2340157) for 1 h at room temperature in the dark. Cells were washed with PBS and stained with 1 µg/µL 4′,6-diamidino-2-phenylindole (DAPI; Sigma-Aldrich). Immunofluorescence analysis was carried out using an Olympus, DP70 equipped with a fluorescent microscope (Reichert-Jung, POLYVER) at 100x magnification. No oil was used, and final image preparation was carried out using ImageJ (1.50i) software.

Mass Spectrometry sample preparation

Preparation of protein eluates for mass spectrometry acquisition was essentially as previously described (6, 7). Briefly, the eluates were dried using a speed vacuum apparatus and re-suspended in 10µL of 20 mM Tris-HCl pH 8.0. Trypsin digestion was carried out using 0.75 µg of trypsin (Sigma) for ~ 15 h at 37°C with mild agitation. An extra 0.25 µg of trypsin was added to each

sample and they were incubated for an additional 3 h. The samples were acidified to a final concentration of 2% acetic acid, desalted using C_{18}StageTips (Thermo Scientific) as per the manufacturer's instructions and stored at -80°C until their acquisition on a mass spectrometer.

Mass spectrometry acquisition using Triple TOF 5600 mass spectrometer

5 µL of each sample, representing 50% of the sample, was directly loaded at 300 nL/min onto a New Objective PicoFrit column (15 cm×0.075 mm I.D; Scientific Instrument Services, Ringoes, NJ) packed with Jupiter 5 µm C_{18} (Phenomenex, Torrance, CA) stationary phase. The peptides were eluted from the column by a gradient generated by an Agilent 1200 HPLC system (Agilent, Santa Clara, CA) equipped with a nano electrospray ion source coupled to a 5600+ Triple TOF mass spectrometer (Sciex, Concord, ON). A 65-min. linear gradient of a 5–35% mixture of 0.1% formic acid injected at 300 nL/min. was used to elute peptides. Data dependent acquisition mode was used in Analyst version 1.7 (Sciex) to acquire mass spectra. Full scan mass spectrum (400 to 1250m/z) were acquired followed by collision-induced dissociation of the twenty most intense ions. A period of 20 s and a tolerance of 100 ppm were set for dynamic exclusion.

Mass spectrometry acquisition using Orbitrap Fusion mass spectrometer

Peptide samples were separated by online reversed-phase (RP) nanoscale capillary liquid chromatography (nanoLC) and analyzed by electrospray mass spectrometry (ESI MS/MS). The experiments were performed with a Dionex UltiMate 3000 nanoRSLC chromatography system (Thermo Fisher Scientific) connected to an Orbitrap Fusion mass spectrometer (Thermo Fisher Scientific) equipped with a nanoelectrospray ion source. Peptides were trapped at 20 ul / min in loading solvent (2% acetonitrile, 0.05% TFA) on a 5mm x 300 µm C18 pepmap cartridge pre-column (Thermo Fisher Scientific) during 5 min. The pre-column was switched online with a self-made 50 cm x 75 µm internal diameter separation column packed with ReproSil-Pur C18-AQ 3-µm resin (Dr. Maisch HPLC) and the peptides were eluted with a linear gradient from 5-40% solvent B (A: 0,1% formic acid, B: 80% acetonitrile, 0.1% formic acid) in 60 min., at 300 nL/min. Mass spectra were acquired using a data dependent acquisition mode using Thermo XCalibur software version 3.0.63. Full scan mass spectra (350 to 1800m/z) were acquired in the orbitrap using an AGC target of 4e5, a maximum injection time of 50 ms and a resolution of 120000. Internal calibration using lock mass on the m/z 445.12003 siloxane ion was used. Each MS scan

was followed by acquisition of fragmentation spectra of the most intense ions for a total cycle time of 3 s (top speed mode). The selected ions were isolated using the quadrupole analyzer in a window of 1.6 m/z and fragmented by Higher energy Collision-induced Dissociation (HCD) with 35% of collision energy. The resulting fragments were detected by the linear ion trap in rapid scan rate with an AGC target of 1E4 and a maximum injection time of 50ms. Dynamic exclusion of previously fragmented peptides was set for a period of 20 s and a tolerance of 10 ppm.

Mass spectrometry acquisition using LTQ mass spectrometer

The re-suspended sample was bomb-loaded in its entirety on the equilibrated column. The column was washed off-line for 10 min. in buffer A and placed in-line with a LTQ mass spectrometer equipped with an Agilent 1100 pump with split flow, and either the Thermo source, or a Proxeon source. Buffer A is 2% acetonitrile (ACN), 0.1% formic acid; buffer B is 98% ACN, 0.1% formic acid. The HPLC gradient program delivered an ACN gradient over 120 min. (1-5% buffer B over 4 min., 5-40% buffer B over 100 min., 40-60% buffer B over 5 min., 60-100% buffer B over 5 min., hold buffer B at 100% 3 min., and 100-0%B in 2 min.). The parameters for Data Dependent Acquisition on the mass spectrometer were: 1 centroid MS (mass range 400-2000) followed by MS/MS on the 5 most abundant ions. General parameters were: activation type = CID, isolation width = 3, normalized collision energy = 32, activation Q = 0.25, activation time = 30 msec, wide band activation. The minimum threshold was 1000, the repeat4count = 1, repeat duration = 30 s, exclusion size list = 500, exclusion duration = 30s, exclusion mass width (by mass) = low 1.2, high 1.5.

Data Dependent Acquisition MS analysis:

Mass spectrometry data were stored, searched and analyzed using the ProHits laboratory information management system (LIMS) platform (8). Within ProHits, Thermo Fisher scientific RAW mass spectrometry files were converted to mzML and mzXML using ProteoWizard (3.0.4468; (9). Within ProHits, AB SCIEXWIFF files were first converted to an MGF formatusing WIFF2MGF converter and to an mzML format using ProteoWizard (v3.0.4468) and the AB SCIEX MS Data Converter (V1.3 beta). The mzML and mzXML files were searched using Mascot (v2.3.02). The spectra were searched with the RefSeq database (version45, January 24th, 2011) acquired from NCBI against a total of 24,770 *T. thermophila* sequences. For TripleTOF files, the

database parameters were set to search for tryptic cleavages, allowing up to two missed cleavage sites per peptide with a mass tolerance of 40 ppm for precursors with charges of 2+ to 4+ and a tolerance of +/- 0.15 amu for fragment ions. For Orbitrap Fusion files, the database parameters were set to search for tryptic cleavages, allowing up to two missed cleavage sites per peptide with a mass tolerance of 12 ppm for precursors with charges of 2+ to 4+ and a tolerance of +/- 0.6 amu for fragment ions. For files analyzed on the LTQ, the charges +1, +2 and+3 were considered, with the parent mass tolerance set at 3 amu and the fragments at 0.6 amu. Deamidated asparagine and glutamine and oxidized methionine were allowed as variable modifications. SAINTexpress version 3.61 (10) was used as a statistical tool to calculate the probability value of each potential protein-protein interaction from background contaminants using default parameters. For the LTQ samples, 39 controls compressed to 20 were used while 15 uncompressed controls were employed for the Triple TOF samples.

Chromatin Immunoprecipitation (ChIP)

90 mL of cells in exponential growth were collected and cross-linked with 2.5 mL of 37% formaldehyde for 30 min. at room temperature and neutralized with 13mL of 2.5M glycine. The chromatin was washed with 10mM Tris pH7.4 followed with cold lysis buffer (50mM Tris pH 8.0, 5mM EDTA, 1% SDS in H_2O plus protease inhibitor (cOmplete™ Protease Inhibitor Cocktail) and 100mM PMSF). The chromatin was fragmented to 400 to 500 base pairs on ice by sonication 10 cycles 25 sec settings duty cycle 30%, output 0.3 (Branson 450 Analog Sonifier). The lysis buffer containing the sonicated chromatin was diluted 10 times using dilution buffer (50mM Tris pH 8.0, 100mM NaCl, 5mM EDTA, 2% Triton X and 0.2% deoxycholate. Chromatin immunoprecipitation was performed using 25uL of packed bead volume M2 agarose beads (Sigma) rotating at 4°C for 4h. The beads were washed one time with each of the following buffers 1X FA, 1X FA plus 500mM NaCl, LiCl buffer and 1X TE. DNA elution started in 2X Proteinase K buffer (20mM Tris pH8.0, 10mM EDTA and 1% SDS in H_2O) at 65°C for 20 minutes and diluted to 1X Proteinase K by H_2O addition. RNase (Fermentas) was added and incubated for 1h at 37°C. Proteinase K (Fermentas) was added and incubated at 42°C for 2 h and 65°C for 8h. The DNA was isolated (Qiagen PCR purification kit) and H_2O was used as elution.

Gene enrichment analysis

The GO enrichment analysis was carried out using g:Profiler (11). We used ChIP-seq bound genes identified by RACS analysis (12) and analyzed this list of genes for enrichment in biological processes and/or molecular functions. Note: RACS identified enriched genes were used only for GO, whereas all other analyses reported in this study were carried out using MACS2 peaks, as detailed in main text methods. We used all annotated *Tetrahymena* genes as a background. The p-value threshold was 0.05 for GO terms to be considered as significantly enriched. To visualize these significant GO terms, we used the enrichment map plug-in in Cytoscape (13).

TCGA data analysis: Differential expression analysis and Kaplan–Meier plots

Gene expression analysis was performed using online tools including 'UALCAN' (http://ualcan.path.uab.edu/index.html) (14), as well as 'Gene expression profiling interactive analysis (GEPIA, http://gepia.cancer-pku.cn/)' (15). These tools provide access to a large collection of data including 'The Cancer Genome Atlas (TCGA)' and the Genotype-Tissue Expression (GTEx) project. Differential expression analysis of *RBBP4* and *RPPB7* in tumor/normal tissue from various cancers was performed. The reported figures (Supplemental Figure S5A) were generated using UALCAN and further modified for visual purposes. For statistical analysis t-tests were performed. Pearson correlations between two given genes were calculated using GEPIA (15). The scatter plot shown in supplemental Figure S6 was constructed using the correlation values (R). Gene dependency scores for RBBP4 and 7 across cancer cell lines was examined using the Dependency Map (DepMap) portal (https://depmap.org/portal/). The DepMap portal encompasses data from RNAi and CRISPR screens across hundreds of cancer cell lines (16, 17).

For survival analysis we used the web-based tool, Kaplan-Meier plotter, to analyze the impact of *RBBP4* and *RBBP7* genes on patient survival using data from 364 liver cancer samples (18). The patients were grouped into high and low groups based on the median expression levels of *RBBP4* and *RBBP7*. Kaplan–Meier survival plots were constructed to compare the two patient groups and calculate the log-rank *p*-value. The correlations between RBBP4/7 expression and patient survival were analyzed using the Kaplan–Meier plotter (http://kmplot.com/analysis/) (18). In addition, survival maps of hazard ratio (heatmaps) were constructed using GEPIA server (http://gepia2.cancer-pku.cn/#index) (15).

Supplementary References

1. Finn,R.D., Coggill,P., Eberhardt,R.Y., Eddy,S.R., Mistry,J., Mitchell,A.L., Potter,S.C., Punta,M., Qureshi,M., Sangrador-Vegas,A., *et al.* (2016) The Pfam protein families database: towards a more sustainable future. *Nucleic Acids Res.*, **44**, D279–D285.

2. Letunic,I. and Bork,P. (2018) 20 years of the SMART protein domain annotation resource. *Nucleic Acids Res.*, **46**, D493–D496.

3. Kumar,S., Stecher,G. and Tamura,K. (2016) MEGA7: Molecular Evolutionary Genetics Analysis Version 7.0 for Bigger Datasets. *Mol. Biol. Evol.*, **33**, 1870–1874.

4. Yang,J., Yan,R., Roy,A., Xu,D., Poisson,J. and Zhang,Y. (2015) The I-TASSER Suite: protein structure and function prediction. *Nat. Methods*, **12**, 7–8.

5. Akematsu,T., Fukuda,Y., Garg,J., Fillingham,J.S., Pearlman,R.E. and Loidl,J. (2017) Post-meiotic DNA double-strand breaks occur in Tetrahymena, and require Topoisomerase II and Spo11. *Elife*, **6**.

6. Saettone,A., Garg,J., Lambert,J.-P., Nabeel-Shah,S., Ponce,M., Burtch,A., Thuppu Mudalige,C., Gingras,A.-C., Pearlman,R.E. and Fillingham,J. (2018) The bromodomain-containing protein Ibd1 links multiple chromatin-related protein complexes to highly expressed genes in Tetrahymena thermophila. *Epigenetics Chromatin*, **11**, 10.

7. Nabeel-Shah,S., Garg,J., Kougnassoukou Tchara,P.E., Pearlman,R.E., Lambert,J.P. and Fillingham,J. (2021) Functional proteomics protocol for the identification of interaction partners in Tetrahymena thermophila. *STAR Protoc.*, **2**.

8. Liu,G., Knight,J.D.R., Zhang,J.P., Tsou,C.-C., Wang,J., Lambert,J.-P., Larsen,B., Tyers,M., Raught,B., Bandeira,N., *et al.* (2016) Data Independent Acquisition analysis in ProHits 4.0. *J. Proteomics*, **149**, 64–68.

9. Kessner,D., Chambers,M., Burke,R., Agus,D. and Mallick,P. (2008) ProteoWizard: open source software for rapid proteomics tools development. *Bioinformatics*, **24**, 2534–6.

10. Teo,G., Liu,G., Zhang,J., Nesvizhskii,A.I., Gingras,A.-C. and Choi,H. (2014) SAINTexpress:

improvements and additional features in Significance Analysis of INTeractome software. *J. Proteomics*, **100**, 37–43.

11. Reimand,J., Arak,T. and Vilo,J. (2011) G:Profiler - A web server for functional interpretation of gene lists (2011 update). *Nucleic Acids Res.*, **39**.
12. Saettone,A., Ponce,M., Nabeel-Shah,S. and Fillingham,J. (2019) RACS: Rapid analysis of ChIP-Seq data for contig based genomes. *BMC Bioinformatics*, **20**.
13. Cline,M.S., Smoot,M., Cerami,E., Kuchinsky,A., Landys,N., Workman,C., Christmas,R., Avila-Campilo,I., Creech,M., Gross,B., *et al.* (2007) Integration of biological networks and gene expression data using Cytoscape. *Nat. Protoc.*, **2**, 2366–82.
14. Chandrashekar,D.S., Bashel,B., Balasubramanya,S.A.H., Creighton,C.J., Ponce-Rodriguez,I., Chakravarthi,B.V.S.K. and Varambally,S. (2017) UALCAN: A Portal for Facilitating Tumor Subgroup Gene Expression and Survival Analyses. *Neoplasia (United States)*, **19**, 649–658.
15. Tang,Z., Li,C., Kang,B., Gao,G., Li,C. and Zhang,Z. (2017) GEPIA: A web server for cancer and normal gene expression profiling and interactive analyses. *Nucleic Acids Res.*, **45**, W98–W102.
16. Meyers,R.M., Bryan,J.G., McFarland,J.M., Weir,B.A., Sizemore,A.E., Xu,H., Dharia,N. V, Montgomery,P.G., Cowley,G.S., Pantel,S., *et al.* (2017) Computational correction of copy number effect improves specificity of CRISPR-Cas9 essentiality screens in cancer cells. *Nat. Genet.*, **49**, 1779–1784.
17. Tsherniak,A., Vazquez,F., Montgomery,P.G., Weir,B.A., Kryukov,G., Cowley,G.S., Gill,S., Harrington,W.F., Pantel,S., Krill-Burger,J.M., *et al.* (2017) Defining a Cancer Dependency Map. *Cell*, **170**, 564-576.e16.
18. Menyhárt,O., Nagy,Á. and Gyórffy,B. (2018) Determining consistent prognostic biomarkers of overall survival and vascular invasion in hepatocellular carcinoma. *R. Soc. Open Sci.*, **5**.
